# Unravelling the relationship between self-efficacy and self-regulated strategies in GenAI-assisted writing of postgraduates: the mediating role of feedback engagement

**DOI:** 10.3389/fpsyg.2026.1786221

**Published:** 2026-07-10

**Authors:** Jiayun Xue, Meihua Chen

**Affiliations:** School of Foreign Languages, Southeast University, Nanjing, China

**Keywords:** feedback engagement, generative AI, L2 writing, self-regulated learning, structural equation modeling, writing self-efficacy

## Abstract

As generative artificial intelligence (GenAI) becomes increasingly embedded in academic writing, understanding how L2 writers engage with AI-generated feedback and regulate their writing processes is critical. While prior research has focused on learners’ perceptions of GenAI and writing outcomes, little is known about the processes through which learners’ beliefs are enacted in GenAI-assisted writing contexts. Drawing on social cognitive theory and self-regulated learning (SRL) frameworks, this study proposes a process-oriented mediation model in which engagement with GenAI feedback links GenAI writing self-efficacy and writing SRL strategies. Survey data were collected from 564 Chinese non-English-major postgraduate students using GenAI for English academic writing. Structural equation modeling revealed that GenAI writing self-efficacy significantly predicted feedback engagement, which in turn predicted writing SRL strategies. The direct relationship between self-efficacy and SRL strategies became non-significant when engagement was included, indicating full mediation, except for the direct significant relationship between self-efficacy and cognitive strategies. The findings position feedback engagement as a central self-regulatory mechanism in GenAI-assisted writing.

## Introduction

Generative artificial intelligence (GenAI) is capable of generating original creative outputs and text that closely mimics human writing, which has gained tremendous momentum and has begun to fundamentally alter instructional approaches for English as a Foreign Language (EFL) writing (Dasborough, 2023). As this technology becomes increasingly embedded in educational settings, it has also brought comprehensive transformations to the broader ecosystem of EFL writing education (Liu et al., 2025). In this study, the term English as a Foreign Language (EFL) is adopted to emphasize the instructional and sociolinguistic context of English learning. The term second language (L2) is used to refer specifically to English learned and used in non-English-speaking contexts. Accordingly, L2 writing and EFL writing are used interchangeably in this study, with both referring to English writing in EFL contexts (Teng and Zhang, 2016). Advances in generative artificial intelligence, have profoundly transformed EFL writing practices. AI-powered feedback generators and interactive writing assistants are increasingly integrated into academic writing contexts, offering learners immediate, personalized, and linguistically rich feedback (Wang et al., 2024). For EFL learners, GenAI provides new opportunities to support idea generation, language accuracy, organization, and revision, thereby reshaping traditional writing processes and learner-technology interactions (Warschauer et al., 2023).

For EFL learners, their self-efficacy in GenAI-supported writing, willingness to engage with feedback, and adoption of writing self-regulated learning (SRL) strategies represent key interconnected psychological factors. These elements shape learners’ composing procedures and further exert an influence on their writing performance (Liu et al., 2025; Chiu, 2024). Yet relevant research that centers on the three constructs simultaneously is still at an early stage. These strategies play a crucial role in enabling learners to manage the complexity of academic writing, particularly in technology-mediated environments (Zimmerman and Kitsantas, 2014).

Within this emerging landscape of GenAI, learner engagement with GenAI feedback has become a crucial factor influencing writing development. Unlike traditional teacher feedback, GenAI feedback is instant, iterative, and available on demand (Dong, 2025). However, how GenAI feedback effects a change is relatively corelated with learners’ engagement (Ellis, 2010). Learners may differ substantially in the extent to which they accept, reflect on, and apply GenAI feedback during writing and revision processes (Han and Hyland, 2015). Moreover, feedback engagement constitutes the critical link between writing self-efficacy beliefs and writing SRL strategies use. Scholars have highlighted unique AI-driven factors, such as prompt revising and feedback assessment (Tam, 2024 Guo and Wang, 2024).

Another key construct in this context is writing self-efficacy, which is closely related to learner’s self-confidence in writing successfully (Pajares and Valiante, 2001). Previous research in L2 writing has proved that writing self-efficacy co-occurs with greater persistence, strategy use, and writing achievement (Teng, 2021). With the increasing adoption of GenAI tools, the notion of GenAI-writing self-efficacy has emerged, referring to learners’ confidence in using GenAI tools effectively to support their writing (Liu and Zhang, 2025). Learners with higher GenAI-writing self-efficacy may be more willing to experiment with GenAI feedback and integrate it into their writing practices (Pack and Maloney, 2023).

Closely related to both feedback engagement and self-efficacy is writing self-regulation, which encompasses learners’ strategic management of cognitive and motivational processes during writing (Zimmerman and Kitsantas, 2014). Writing self-regulation refers to the overall capacity to monitor and control one’s writing process, whereas self-regulated writing strategies represent the concrete, context-specific tactics employed to enact such regulatory capacity. In L2 writing research, such strategies have been conceptualized as observable, operationalizable behaviors that reflect underlying self-regulation (Teng and Zhang, 2016). Self-regulated writing strategies enable learners to plan and adjust their writing behaviors. Self-regulated writing strategies enable learners to plan, monitor, evaluate, and adjust their writing behaviors. In technology-enhanced learning environments, effective self-regulation becomes even more essential, as learners must autonomously decide how and when to use digital tools, including GenAI systems (Wang, 2024). Self-efficacy motivationally engages learners, which facilitates learners in gaining firmer command over SRL strategies (Teng, 2021). In GenAI-assisted writing contexts, learners with higher GenAI-writing self-efficacy may be more inclined to actively engage with GenAI feedback and to deploy a wider range of writing SRL strategies (Liu et al., 2025). Few studies have examined the interactions among GenAI writing self-efficacy, GenAI writing self-regulated strategies, and their influences on feedback engagement for L2 learners, particularly in the generative AI context (Huang and Mizumoto, 2025).

Although previous research has separately examined self-efficacy, feedback engagement, and SRL strategies, few studies have integrated them into a unified model within GenAI-assisted EFL writing contexts, especially for graduate students. It remains unclear how GenAI writing self-efficacy is associated with self-regulation and whether feedback engagement serves as a mediator. Given the unique features of GenAI tools, traditional relationships among these variables may be reshaped. Therefore, this study aims to establish a process-oriented model to explore the relationships among GenAI writing self-efficacy, feedback engagement, and SRL strategies, which will provide both theoretical and pedagogical insights into AI-mediated L2 writing instruction.

## Literature review

### Writing self-efficacy

Self-efficacy is a context-sensitive construct referring to learners’ beliefs in their ability to accomplish a specific task successfully (Bandura, 1993; Kim et al., 2015). In writing research, writing self-efficacy has been defined as individuals’ judgments of their ability to accomplish writing tasks by drawing on composition, grammatical, and mechanical skills (Pajares and Valiante, 2001). Extensive research has established writing self-efficacy as a key motivational factor influencing learners’ persistence, strategy use, and writing performance (Teng, 2021).

In GenAI-assisted writing contexts, writing self-efficacy acquires new characteristics. According to social cognitive theory (Bandura, 1997), learners’ mastery experiences and physiological states, help explain the mixed effects of GenAI on EFL writers’ perceived writing competence. First, GenAI-generated outputs can provide learners with successful writing experiences by offering linguistically polished and contextually appropriate suggestions, which may enhance writing self-efficacy (Pack and Maloney, 2023). However, such mastery experiences are contingent on learners’ ability to formulate effective prompts; without sufficient prompt literacy, GenAI feedback may appear generic or misaligned with writing goals, undermining confidence (Lee and Palmer, 2025).

GenAI supports vicarious learning by synthesizing linguistic patterns and discourse conventions from large-scale textual data. Yet, the presence of biased, oversimplified, or fabricated information in AI-generated feedback may increase learners’ cognitive load and uncertainty, potentially diminishing writing self-efficacy (Spennemann, 2025). Third, GenAI interactions can elicit both positive and negative emotional responses. While adaptive and responsive AI feedback may foster enjoyment and reassurance (Lin and Ng, 2024), concerns about authorship, identity, and dependence on AI may evoke anxiety and reduce learners’ perceived writing competence (Alessandro et al., 2025; Jabali et al., 2025).

Empirical findings regarding the effects of GenAI on writing self-efficacy remain inconsistent. Meta-analytic and empirical evidence suggests that factors such as feedback type, intervention duration, learner proficiency, and instructional context moderate these effects (Xue, 2024). Methodological limitations further contribute to these inconsistencies, as most existing studies rely on descriptive or outcome-oriented approaches, with relatively few employing process-oriented, variable-centered designs such as structural equation modeling to examine underlying mechanisms (Huang and Mizumoto, 2025).

Taken together, prior research indicates that writing self-efficacy in GenAI-assisted contexts cannot be understood solely as a direct outcome of AI use. Rather, it is necessary to examine how learners’ self-efficacy beliefs are enacted during writing through engagement with GenAI feedback and strategic regulation of the writing process (Liu and Zhang, 2025).

### Engagement with generative AI feedback

Engagement with feedback refers to learners’ engagement in feedback processing and response during learning tasks. In L2 writing research, feedback engagement has been conceptualized as a multidimensional construct encompassing cognitive, behavioral, and affective dimensions (Ellis, 2010; Han and Hyland, 2015). Rather than passively receiving feedback, engaged writers interpret feedback in relation to task goals, make decisions about adoption or rejection, and regulate their revision behavior accordingly (Zhang and Hyland, 2022).

In GenAI-assisted writing contexts, feedback engagement takes on heightened importance due to the distinctive characteristics of AI-generated feedback. Unlike teacher or peer feedback, GenAI feedback is learner-initiated, iterative, and highly contingent on prompt formulation (Dong, 2025). As a result, learners are required not only to evaluate feedback quality and relevance but also to continuously refine their interaction with the tool (Tam, 2024). Engagement thus becomes an ongoing process of negotiation between learner intentions and AI-generated responses (Koltovskaia et al., 2024).

Recent studies suggest that learners vary considerably in how they engage with GenAI feedback. Some learners strategically compare AI suggestions with their own drafts, selectively integrating feedback to support meaning development and rhetorical clarity, whereas others adopt feedback uncritically or rely on surface-level revisions (Bai and Wang, 2021; Huang and Renandya, 2020). Such variation indicates that the effectiveness of GenAI feedback cannot be attributed to the tool itself but rather to learners’ engagement practices (Rad et al., 2024).

Importantly, engagement with GenAI feedback is closely linked to learners’ motivational beliefs (Derakhshan and Fathi, 2023). Learners with higher confidence in their ability to use GenAI tend to showcase more willingness to experiment with prompts, evaluate feedback critically, and persist through multiple revision cycles (Liu and Zhang, 2025). This suggests that engagement may serve as the mechanism through which motivational resources are mobilized during AI-assisted writing, linking beliefs about GenAI use to concrete regulatory actions (Reeve et al., 2020). Although motivational beliefs and feedback engagement may correlate dynamically, in social cognitive theory, self-efficacy functions as an antecedent motivational resource that precedes and facilitates engagement behaviors. Thus, the present model establishes a directional path from self-efficacy to feedback engagement, which is consistent with theoretical and empirical conventions in SRL research.

### Writing self-regulated learning strategies

Self-regulated learning (SRL) in writing refers to learners’ ability to monitor, and evaluate their writing processes through the strategic management of cognitive or behavioral resources (Zimmerman and Kitsantas, 2014). Writing SRL strategies typically include goal setting, strategic planning, self-monitoring, feedback use, and reflective evaluation, all of which are essential for successful academic writing, particularly in second language contexts (Teng and Zhang, 2016).

From a social cognitive perspective, SRL is not a fixed trait but a dynamic process shaped by task demands and learning environments (Bandura, 1997). In technology-mediated writing contexts, learners are required to coordinate multiple sources of feedback and make autonomous decisions about strategy use (Wang, 2024). GenAI-assisted writing amplifies these demands by providing abundant and immediate feedback, thereby increasing both opportunities for regulation and the risk of over-reliance.

Emerging research suggests that GenAI empowers learners in terms of brainstorming, refining writing tones, and polishing up logical reasoning. However, the effectiveness of such assistance is contingent upon learners’ competence in managing tool engagement. Without effective self-regulation, learners may engage in superficial revisions, outsource decision-making to AI, or disengage from higher-level writing goals.

Crucially, writing SRL strategies are unlikely to be directly activated by access to GenAI alone. Instead, they are enacted through learners’ engagement with feedback during the writing process (Teng and Zhang, 2022). Engaged interaction with GenAI feedback, such as evaluating suggestions against rhetorical goals or reflecting on alternative revisions, creates opportunities for planning, monitoring, and evaluation (Han and Hyland, 2015). From this perspective, engagement with GenAI feedback can be understood as the proximal process through which self-regulated writing behavior is realized in AI-assisted contexts (Reeve and Tseng, 2011).

## Methodology

### Research questions

Building upon the research orientation provided by previous literature, this study aims to further explore the relationships among Chinese postgraduate students’ GenAI assisted writing self-efficacy, GenAI-feedback engagement and self-regulation strategies in the context of GenAI L2 assisted writing. The specific research questions are as follows:

1. What is the relationship among Chinese postgraduate students’ GenAI assisted writing self-efficacy, GenAI-feedback engagement and writing self-regulation strategies in the context of GenAI assisted writing?
2. To what extent do GenAI-assisted writing self-efficacy and GenAI-feedback engagement directly influence Chinese postgraduate students’ writing self-regulation strategies in GenAI-assisted EFL writing contexts?
3. To what extent does GenAI-feedback engagement mediate the relationship between Chinese postgraduate students’ GenAI assisted writing self-efficacy and writing self-regulation strategies in the context of GenAI assisted writing?

Given what has been stated above, *Hypotheses* of the research are stated as follows:

*H1a:* GenAI-feedback engagement mediates the relationship between GenAI assisted writing self-efficacy and cognitive writing self-regulation strategies.

*H1b:* GenAI-feedback engagement mediates the relationship between GenAI assisted writing self-efficacy and metacognitive writing self-regulation strategies.

*H1c:* GenAI-feedback engagement mediates the relationship between GenAI assisted writing self-efficacy and social writing self-regulation strategies.

*H1d:* GenAI-feedback engagement mediates the relationship between GenAI assisted writing self-efficacy and motivational writing self-regulation strategies.

*H2a:* GenAI-feedback engagement positively predicts cognitive writing self-regulation strategies.

*H2b:* GenAI-feedback engagement positively predicts metacognitive writing self-regulation strategies.

*H2c:* GenAI-feedback engagement positively predicts social writing self-regulation strategies.

*H2d:* GenAI-feedback engagement positively predicts motivational writing self-regulation strategies.

**Figure 1:**
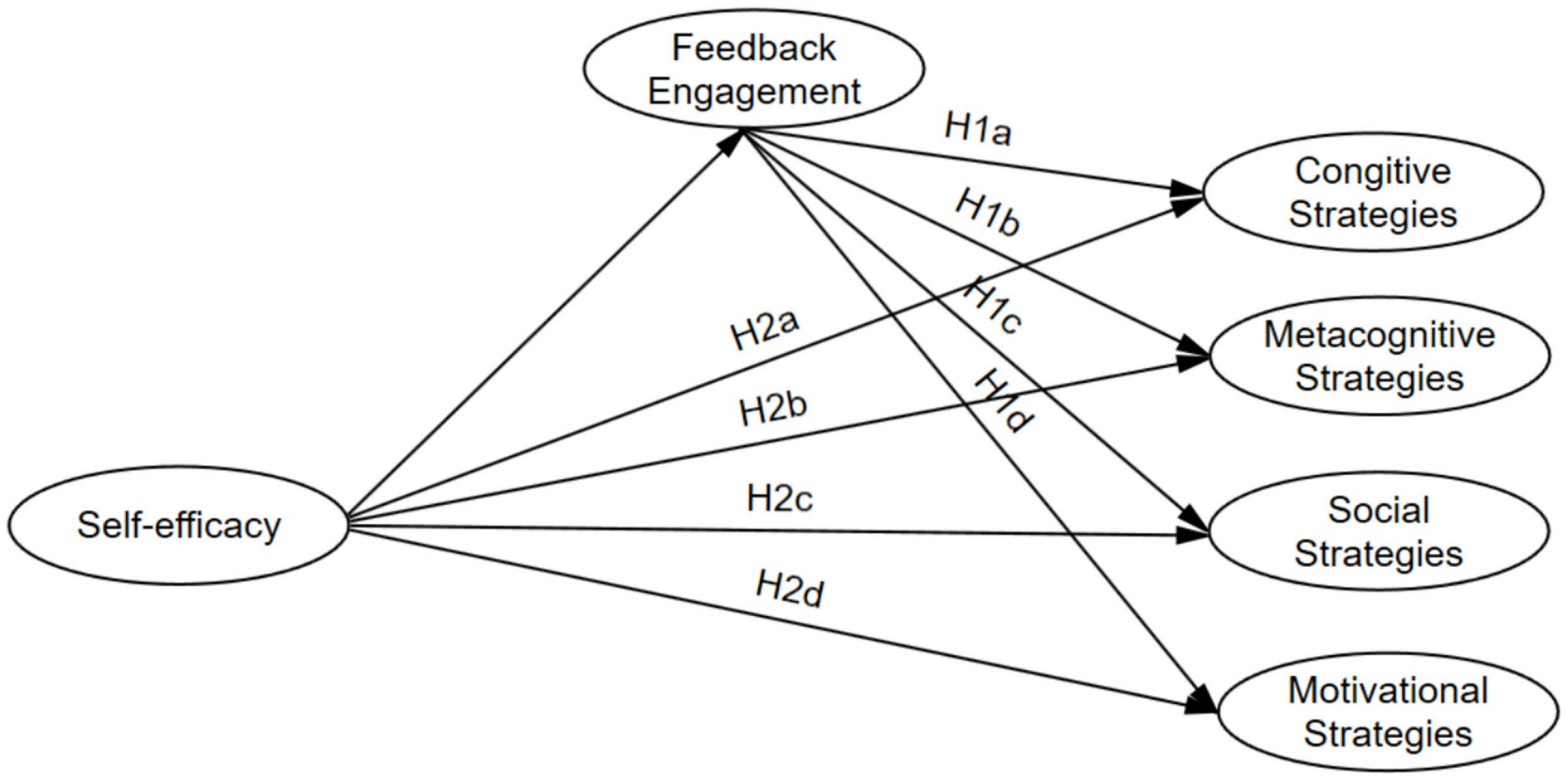
The hypothesized model.

The hypothesized model is illustrated in Figure 1.

### Participants

Participants in the study were non-English majors recruited from eleven comprehensive universities across different regions of China, ensuring diversity in educational backgrounds and demographic representation (see Table 1). Participants were recruited using a convenience sampling approach, primarily through course-based invitations and academic networks. To enhance sample heterogeneity and improve the potential representativeness of the data, participants were drawn from multiple institutions and diverse disciplinary backgrounds, all of whom were non-English-major postgraduate students. The inclusion of multiple universities helps mitigate institutional bias and supports cautious generalization of the findings to similar EFL postgraduate populations in higher education (Dörnyei and Taguchi, 2009). A total of 564 questionnaires were collected, of which 43 were removed due to inconsistent responses, duplicate submissions, lack of reported GenAI writing experience, or excessive missing data. The final valid sample size was 521, which meets the generally accepted criteria for SEM (*n* ≥ 200–500) and ensures adequate statistical power for latent variable analysis (Kline, 2015).

**Table 1:** Demographic information (*N* = 521).

Characteristics	Category	*n*	Percentage
Gender distribution	Male	226	43.38
	Female	295	56.62
Educational background	Master’s student	320	61.42
	Doctoral student	201	38.58
Age	22–25 years	308	59.12
	26 or above	213	40.88
Academic major	Arts and humanities	253	48.56
	STEM	268	51.44
Length of learning English	Less than 10 years	0	0
	10 years or above	521	100
Frequency of GAI use	Several times a day	50	9.60
	Several times a week	131	25.14
	Once a week	72	14.01
	Once every 2 weeks	33	6.33
	Once a month or longer	235	45.10

This study was conducted in line with the ethical principles outlined in the Declaration of Helsinki and approved by the institutional review board. Prior to data collection, participants were briefed on the research rationale, procedures, and unconditional rights to terminate participation at any stage, with written informed consent signed and retrieved from all individuals.

### Research instruments

This research utilizes three scales to form a questionnaire, which is adapted from existing validated instruments (Dong, 2025; Liu and Zhang, 2025; Teng and Zhang, 2016). It comprises four sections, including a demographic part to document the participants’ background information (e.g., age, gender, major, CET-4 score, GenAI use experience) and three scales measuring GenAI-assisted writing self-efficacy, GenAI-feedback engagement, and writing self-regulation strategies. The three scales are described in detail below.

#### GenAI assisted writing self-efficacy scale

Adapted from Liu and Zhang (2025), this scale measures GenAI-assisted writing self-efficacy across four dimensions: (1) assistance of GenAI for writing (4 items); (2) anthropomorphic interaction with GenAI for writing (4 items); (3) comfort with GenAI for writing (4 items); (4) technological skills of GenAI-assisted writing (3 items). These dimensions, respectively, assess the perceived helpfulness of GenAI for writing tasks, the degree of anthropomorphism in interaction, emotional awareness during interaction, and confidence in using GenAI for writing. Liu and Zhang (2025) adapted and developed the scale by organizing focus group interviews and existing instruments (Liu et al., 2025; Wang et al., 2024; Zhang et al., 2023). The 15-item scale used a 6-point Likert scale (1 = strongly disagree to 6 = strongly agree). In the formal study, it demonstrated excellent internal consistency (Cronbach’s *α* = 0.931) and model fit (*χ*^2^/df = 2.341, GFI = 0.928, CFI = 0.945, TLI = 0.937, RMSEA = 0.048, SRMR = 0.056), leading to no item removal (Wen et al., 2004).

#### GenAI-feedback engagement scale in L2 writing

Students’ engagement with GenAI feedback was assessed using Li’s (2025) validated scale, originally developed for ChatGPT feedback. It comprises four dimensions: cognitive engagement (6 items), behavioral engagement (9 items), emotional engagement (3 items), and ethical engagement (4 items). Items were rephrased to refer to GenAI feedback broadly (e.g., “I recognize the strengths and weaknesses of my writing based on GenAI feedback”). The scale was translated into Chinese following back-translation procedures (Dörnyei and Taguchi, 2009). Responses were collected on a 5-point Likert scale (1 = strongly disagree to 5 = strongly agree). The scale showed good internal consistency in the formal study (Cronbach’s *α* = 0.945) after the removal of Q3 with satisfactory model fit (χ^2^/df = 1.518, GFI = 0.868, CFI = 0.946, TLI = 0.932, RMSEA = 0.064, SRMR = 0.0632).

#### Writing strategies for self-regulated learning scale

The Writing Strategies for Self-regulated Learning Questionnaire (WSSRLQ) (Teng and Zhang, 2016) was used to assess SRL strategy use. This 40-item scale, based on socio-cognitive and SRL theories (Bandura, 1999; Zimmerman, 1989), includes four categories: cognitive strategy (9 items), metacognitive strategy (9 items), social behavioral strategy (7 items), and motivational regulation strategy (15 items). Items were adapted to the GenAI context (e.g., “when using GenAI-assisted writing, I will check and correct grammar mistakes according to GenAI feedback”). A 6-point Likert scale was used (1 = strongly disagree to 6 = strongly agree). Internal consistency was excellent (Cronbach’s *α* = 0.952). In the formal study, the items (Q1, Q5, Q11, Q12, Q24, Q34) were removed, and confirmatory factor analysis indicated a good model fit (χ^2^/df = 2.341, GFI = 0.928, CFI = 0.945, TLI = 0.937, RMSEA = 0.048, SRMR = 0.056).

### Procedure

The data collection process was conducted in three phases over a period of 2 months (from March to April 2025) to ensure the validity and reliability of the data.

#### Pilot study

Prior to the formal data collection, a pilot study was conducted with 164 non-English-major postgraduate students from the same universities as the formal study participants. The purpose of the pilot study was to test the clarity, comprehensibility, and appropriateness of the questionnaire items, as well as to assess the internal consistency of the scales. The 164 pilot participants were recruited from the same target population (non-English major postgraduates) as the formal sample to ensure representativeness for instrument validation and item refinement. Based on the feedback from the pilot study participants, minor revisions were made to some items to improve their clarity and readability, such as rephrasing ambiguous expressions and simplifying complex sentence structures.

#### Formal data collection

The formal questionnaire was distributed online via Wenjuanxing. Before participating in the survey, participants were provided with an informed consent form that explained the purpose of the study, the voluntary nature of participation, the anonymity of responses, and the right to withdraw from the study at any time without penalty. Participants who agreed to participate were directed to complete the questionnaire. The questionnaire took approximately 15–20 min to complete. To encourage honest responses, participants were informed that their responses would be used solely for research purposes and would be kept strictly confidential. A total of 564 questionnaires were collected during the formal data collection phase.

#### Data screening and cleaning

After the formal data collection, the collected data were screened and cleaned to ensure data quality. First, attention check items were used to identify inattentive responses. Participants who failed to answer the attention check items correctly (e.g., selecting the wrong option as instructed) were excluded. Second, response completion time was examined. Questionnaires completed in less than 3 min were considered invalid and excluded, as they were deemed to reflect hasty or inattentive responding. Third, missing data were handled. The researchers excluded questionnaires with more than 10% missing items. In terms of questionnaires consisting of less than 10% missing items, the mean imputation method was used to fill in the missing values. Fourth, duplicate responses (identified by the same IP address or identical responses to all items) were excluded. After the data screening and cleaning process, 521 valid questionnaires were retained for further analyses.

### Data analysis

The collected data were analyzed using SPSS 26.0 and AMOS 24.0. The following statistical analyses were performed:

#### Descriptive statistics

Descriptive statistics (mean, standard deviation, skewness, kurtosis) were calculated to examine the distribution of the main variables.

#### Correlation analysis

Pearson correlation analysis was conducted to examine the relationships among GenAI-assisted writing self-efficacy, GenAI-feedback engagement, and the four dimensions of writing self-regulation strategies.

#### Confirmatory factor analysis (CFA)

CFA was performed to verify the construct validity of the three scales. The model fit indices, including *χ*^2^/df, GFI, CFI, TLI, RMSEA, and SRMR, were used to evaluate the fitness of the measurement models. According to Wen et al. (2004), *χ*^2^/df between 1 and 3, GFI, CFI, and TLI higher than 0.90, and RMSEA and SRMR less than 0.08 are considered a good fit.

#### Structural equation modeling (SEM)

**Figure 2:**
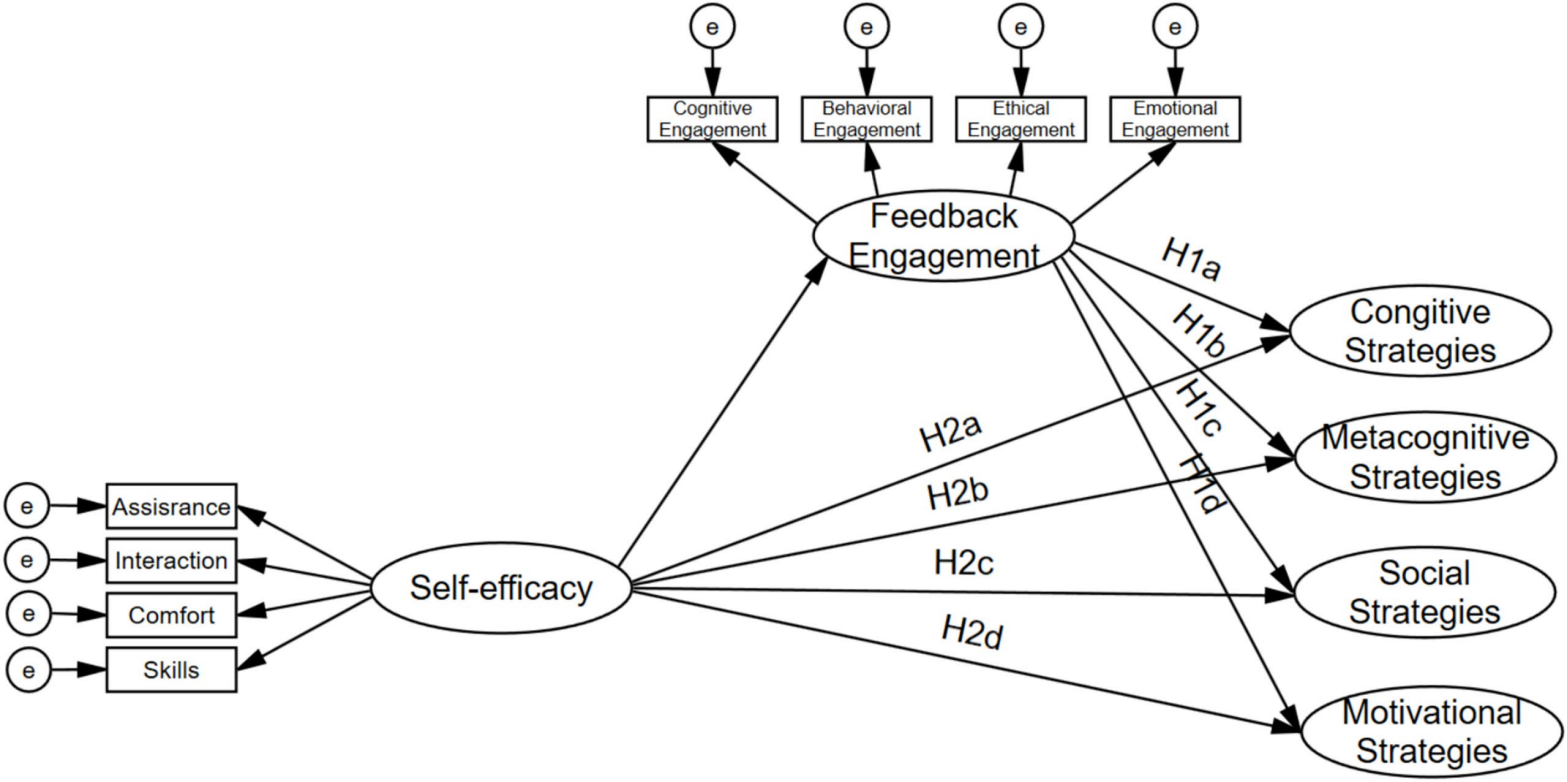
The hypothesized model with item parceling method.

**Figure 3:**
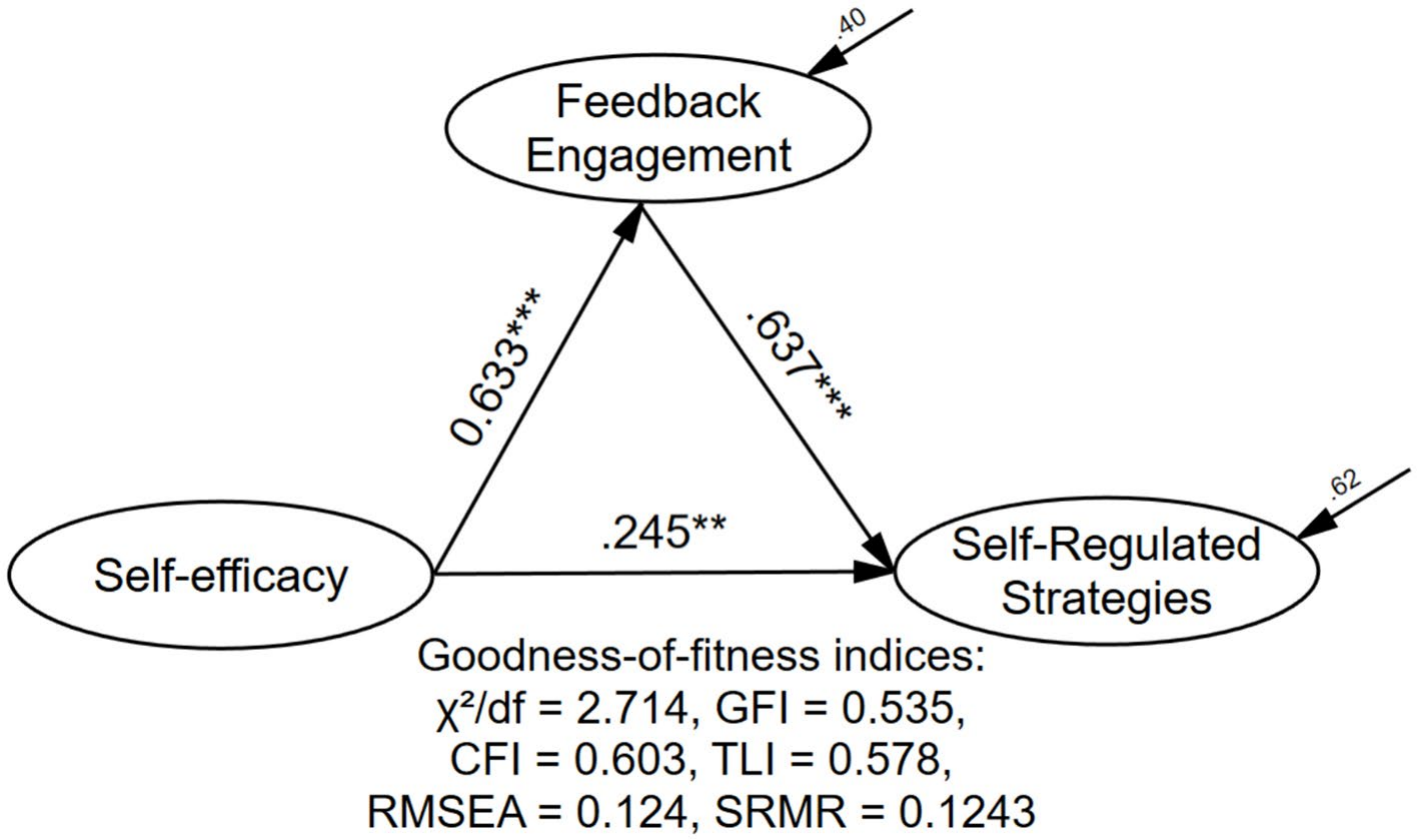
The simplified model (*p < 0.05, **p < 0.01, ***p < 0.001).

SEM was used to test the hypothesized mediation model. Critical data such as the standardized path coefficients and standard errors was examined to determine the direct effects among the variables. Item parceling was employed to improve model fit and reduce measurement error, given the complexity of the multi-dimensional variables in this study, in terms of writing self-efficacy (in four dimension-specific parcels) and feedback engagement (in four dimension-specific parcels). Feedback engagement is conceptualized as a higher-order model because cognitive, behavioral, emotional, and ethical engagement reflect a single underlying regulatory process. This structure enhances model parsimony, stability, and interpretability in SEM analysis. The way of parceling is illustrated in Figure 2. This approach enhances indicator quality by increasing communality and reducing random error, while also simplifying the model for more stable estimates (Wu and Wen, 2011). Potential estimation bias is generally minimal and correctable, making it a practical strategy despite a relatively slight reduction in sensitivity (see Figure 3).

#### Mediation effect test

The bootstrapping method was used to test the mediation effect of GenAI-feedback engagement. A bootstrap sample of 5,000 was generated, and the 95% confidence interval (CI) was used to determine the significance of the indirect effects. If the 95% CI does not include zero, the mediation effect is considered significant (Hayes, 2013).

## Results

### Descriptive statistics and correlation analysis

Table 2 presents the descriptive statistics (mean, standard deviation, skewness, kurtosis) of the main variables. The results show that the skewness and kurtosis values of all variables are within the acceptable range (|skewness| < 2, |kurtosis| < 7), indicating that the data are approximately normally distributed (Kline, 2015), which is suitable for SEM analysis. It also presents the results of the Pearson correlation analysis among the main variables. The results show that GenAI-assisted writing self-efficacy is significantly positively correlated with GenAI-feedback engagement (*r* = 0.503, *p* < 0.001) and all four dimensions of writing self-regulation strategies (CS: *r* = 0.595, *p* < 0.001; McS: *r* = 0.475, *p* < 0.001; SS: *r* = 0.438, *p* < 0.001; MoS: *r* = 0.333, *p* < 0.001). GenAI-feedback engagement is also significantly positively correlated with all four dimensions of writing self-regulation strategies (CS: *r* = 0.749, *p* < 0.001; McS: *r* = 0.666, *p* < 0.001; SS: *r* = 0.570, *p* < 0.001; MoS: *r* = 0.579, *p* < 0.001).

**Table 2:** Descriptive statistics of the main variables.

Variable	Mean	SD	Skewness	Kurtosis	1	2	3	4	5
1 WSE	4.23	0.823	0.092	0.287					
2 FE	3.98	0.62	0.409	−0.725	0.503^***^				
3 CS	4.31	0.59	0.025	−0.741	0.595^***^	0.749***			
4 McS	4.15	0.61	0.098	−0.737	0.475***	0.666***	0.664***		
5 SS	3.87	0.65	−0.034	0.321	0.438***	0.570***	0.545***	0.694***	
6 MoS	3.92	0.63	−0.102	−0.036	0.333***	0.579***	0.570***	0.670***	0.675***

WSE, GenAI-assisted writing self-efficacy; FE, GenAI feedback engagement; CS, cognitive strategies; McS, metacognitive strategies; SS, social strategies; MoS, motivational strategies. *p < 0.05, **p < 0.01, ***p < 0.001.

### Measurement model fit

**Figure 4:**
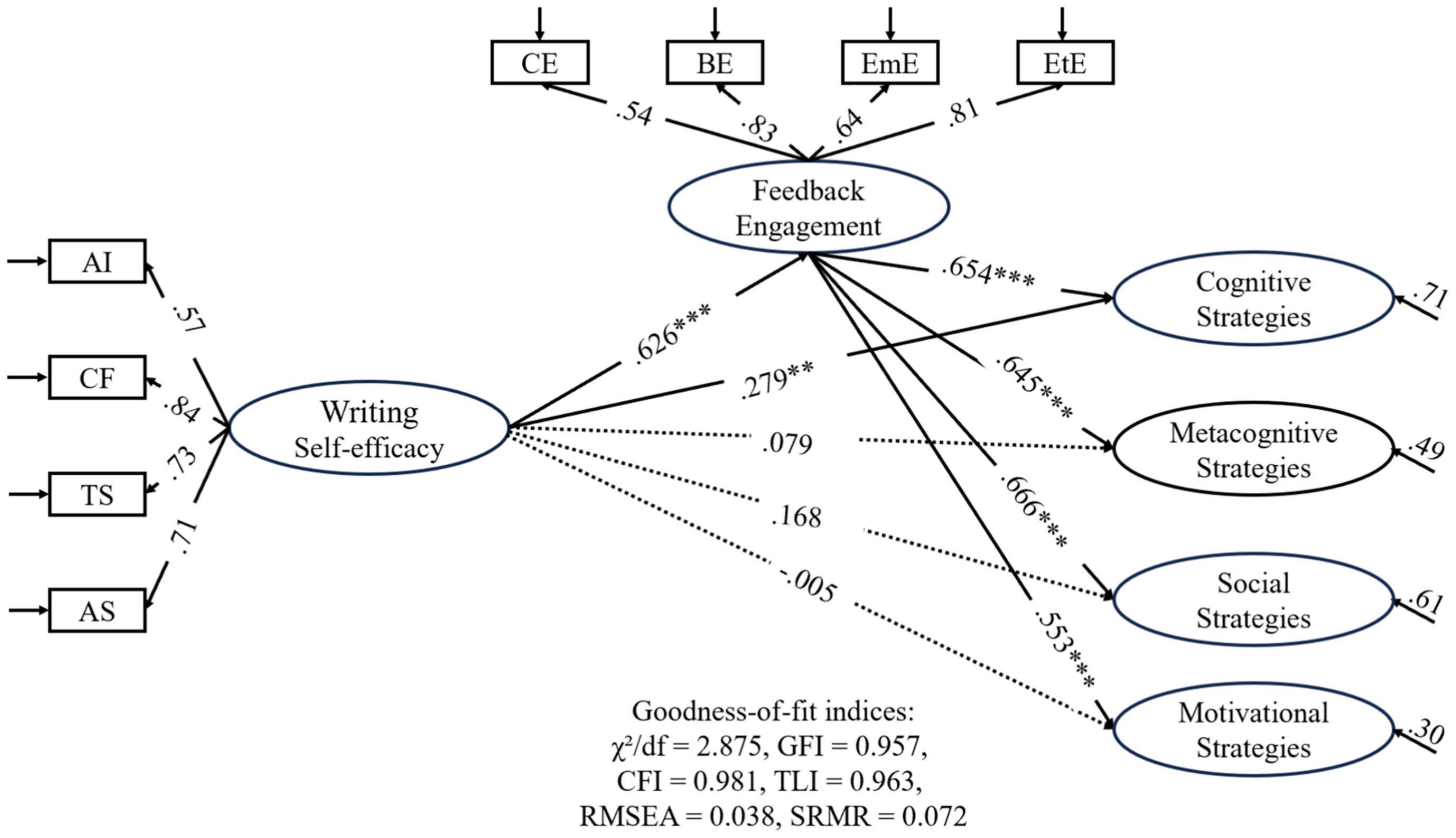
The model of Chinese postgraduates’ GenAI feedback engagement, GenAI self-efficacy, and various self-regulation strategies with standardized estimates. AI, anthropomorphic interaction with GenAI for writing; CF, comfort with GenAI for writing; TS, technological skills of GenAI-assisted writing; AS, assistance of GenAI for writing; CE, cognitive engagement; BE, behavioral engagement; EmE, emotional engagement; EtE, ethical engagement.

The structural equation model (Figure 4) was used to test the hypothesized relationships among GenAI-assisted writing self-efficacy, GenAI-feedback engagement, and writing self-regulation strategies. The results showed that the structural model fit indices were *χ*^2^/df = 2.875, GFI = 0.957, CFI = 0.981, TLI = 0.963, RMSEA = 0.038, SRMR = 0.072, all meeting the recommended standards (Wen et al., 2004), indicating that the model had a good fit to the data. All these indices met the recommended standards (Wen et al., 2004), indicating that the measurement model had a good fit and the construct validity of the scales was acceptable.

### Structural model fit and direct effects

Table 3 presents the standardized path coefficients, and significance levels of the direct effects. As shown in Table 4, GenAI-assisted writing self-efficacy significantly predicted GenAI-feedback engagement (*β* = 0.626, SE = 0.079, CR = 6.603, *p* < 0.001), supporting the positive relationship between the two variables. Regarding the direct effects of GenAI-assisted writing self-efficacy on writing self-regulation strategies, GenAI-assisted writing self-efficacy had a significant positive effect on cognitive strategies (*β* = 0.279, SE = 0.099, CR = 2.812, *p* = 0.005), but its direct effects on metacognitive strategies (*β* = 0.079, SE = 0.130, CR = 0.717, *p* = 0.473), social strategies (*β* = 0.168, SE = 0.121, CR = 1.810, *p* = 0.070), and motivational strategies (*β* = −0.005, SE = 0.163, CR = 4.536, *p* = 0.870) were not statistically significant. In contrast, GenAI-feedback engagement exerted strong and significant positive effects on all four types of writing self-regulation strategies, including cognitive strategies (*β* = 0.654, SE = 0.133, CR = 6.327, *p* < 0.001), metacognitive strategies (*β* = 0.645, SE = 0.169, CR = 5.435, *p* < 0.001), social strategies (*β* = 0.666, SE = 0.145, CR = 7.207, *p* < 0.001), and motivational strategies (*β* = 0.553, SE = 0.163, CR = 4.536, *p* < 0.001). These results support hypotheses H2a, H2b, H2c, and H2d.

**Table 3:** Results of mediation effects.

Path	Parameter	STD.	Bias-corrected 95% CI			Ratio (%)
		Estimate	Lower	Upper	*p*	
GenAI writing self-efficacy → GenAI feedback engagement → Cognitive strategies	Direct effect	0.279	0.057	0.500	0.019	59.51
	Indirect effect	0.410	0.297	0.667	0.000	
	Total effect	0.689	0.534	0.952	0.000	
GenAI writing self-efficacy → GenAI feedback engagement→ Metacognitive strategies	Direct effect	0.079	−0.271	0.449	. 590	100
	Indirect effect	0.404	0.256	0.831	0.003	
	Total effect	0.483	0.337	0.839	0.000	
GenAI writing self-efficacy → GenAI feedback engagement → Social strategies	Direct effect	0.168	−0.092	0.534	0.150	100
	Indirect effect	0.418	0.335	0.887	0.002	
	Total effect	0.585	0.538	1.081	0.000	
GenAI writing self-efficacy → GenAI feedback engagement → Motivational strategies	Direct effect	−0.005	−0.363	0.395	0.967	100
	Indirect effect	0.346	0.151	0.760	0.005	
	Total effect	0.341	0.161	0.651	0.001	

**Table 4:** The internal consistency reliability and convergent validity.

Variable	Item	Reliability (Cronbach’s *α*)	AVE	CR
AS	4	0.862	0.562	0.836
AI	4	0.911	0.721	0.912
CF	4	0.833	0.580	0.805
TS	3	0.940	0.661	0.854
CE	5	0.890	0.912	0.536
BE	9	0.880	0.842	0.519
EtE	4	0.807	0.806	0.510
EmE	3	0.744	0.762	0.518
CS	7	0.822	0.821	0.608
McS	7	0.869	0.803	0.508
SS	6	0.831	0.821	0.605
MoS	14	0.928	0.882	520

AS, assistance of GenAI for writing for writing; AI, anthropomorphic interaction with GenAI; CF, comfort with GenAI for writing; TS, technological skills of GenAI-assisted writing; CE, cognitive engagement; BE, behavioral engagement; EtE, ethical engagement; EmE, emotional engagement; CS, cognitive strategies; McS, metacognitive strategies; SS, social strategies; MoS, motivational strategies.

### Mediation effects

The bootstrapping method was used to test the mediation effect of GenAI-feedback engagement on the relationship between GenAI-assisted writing self-efficacy and writing self-regulation strategies. Table 2 presents the results of the mediation effect analysis.

For cognitive strategies, the results showed a significant direct effect of GenAI-assisted writing self-efficacy [*β* = 0.279, 95% CI (0.057, 0.500), *p* = 0.019] as well as a significant indirect effect through GenAI-feedback engagement [*β* = 0.410, 95% CI (0.297, 0.667), *p* < 0.001]. The total effect was also significant [*β* = 0.689, 95% CI (0.534, 0.952), *p* < 0.001], indicating partial mediation. Thus, hypothesis H1a was supported.

In terms of metacognitive strategies, the direct effect of GenAI-assisted writing self-efficacy was not significant [*β* = 0.079, 95% CI (−0.271, 0.449), *p* = 0.590], whereas the indirect effect via GenAI-feedback engagement was significant [*β* = 0.404, 95% CI (0.256, 0.831), *p* = 0.003]. The total effect was significant [*β* = 0.483, 95% CI (0.337, 0.839), *p* < 0.001], suggesting full mediation. Thus, hypothesis H1b was supported.

A similar pattern was observed for social strategies. The direct effect was non-significant [*β* = 0.168, 95% CI (−0.092, 0.534), *p* = 0.150], while the indirect effect through GenAI-feedback engagement was significant [*β* = 0.418, 95% CI (0.335, 0.887), *p* = 0.002]. The total effect remained significant [*β* = 0.585, 95% CI (0.538, 1.081), *p* < 0.001], again indicating full mediation. Thus, hypothesis H1c was supported.

For motivational strategies, GenAI-assisted writing self-efficacy did not show a significant direct effect [*β* = −0.005, 95% CI (−0.363, 0.395), *p* = 0.967]. However, the indirect effect via GenAI-feedback engagement was significant [*β* = 0.346, 95% CI (0.151, 0.760), *p* = 0.005], and the total effect was also significant [*β* = 0.341, 95% CI (0.161, 0.651), *p* = 0.001], supporting a pattern of full mediation. Thus, hypothesis H1d was supported.

The mediation effect size indicates that GenAI feedback engagement serves as a predominant mechanism in the model, with effect sizes ranging from 59.51 to 100%. Specifically, it partially mediates the path to cognitive strategies, accounting for 59.51% of the total effect. For metacognitive, social, and motivational strategies, the results demonstrate full mediation, as the direct relationships between writing self-efficacy and these outcomes become non-significant in the presence of the mediator.

## Discussion

This study set out to examine how graduate EFL writers’ beliefs about using generative AI are translated into self-regulated writing behavior by testing a mediation model that positions GenAI feedback engagement as the key mechanism linking GenAI writing self-efficacy and writing SRL strategies. The findings provide important theoretical and practical insights into the dynamics of GenAI-assisted L2 writing, which are discussed in detail below.

### Feedback engagement as a translational mechanism

The most salient finding is that GenAI feedback engagement serves as a crucial mediator linking GenAI writing self-efficacy to learners’ use of self-regulated writing strategies. This extends prior research on feedback engagement in traditional and digital writing contexts (Ekholm et al., 2015; Ellis, 2010; Han and Hyland, 2015; Zhang and Hyland, 2022) by demonstrating its central role in GenAI-mediated environments. Specifically, GenAI writing self-efficacy significantly predicts feedback engagement, which in turn positively predicts all four dimensions of writing SRL strategies. This supports social cognitive theory that self-efficacy beliefs predict behavior through motivational and behavioral mechanisms (Bandura, 1997), with feedback engagement acting as the critical interface between beliefs and action in GenAI-assisted writing.

The mediation patterns are noteworthy. For metacognitive, social, and motivational strategies, GenAI feedback engagement fully mediates the relationship between self-efficacy and SRL strategy use. This means self-efficacy’s influence is entirely transmitted through engagement with GenAI feedback. For cognitive strategies, mediation is partial, indicating both direct and indirect effects of self-efficacy. This difference may stem from the nature of cognitive strategies, which relate to basic writing skills (e.g., grammar, vocabulary) deployable regardless of feedback engagement (Graham et al., 2024). In contrast, metacognitive (e.g., planning), social (e.g., collaborating), and motivational (e.g., goal setting) strategies depend more on active engagement with external feedback.

This aligns with Koltovskaia et al. (2024), who found engagement with ChatGPT feedback associated with strategy use in revision. It also supports Reeve et al. (2020) that engagement orchestrates self-regulated learning in technology-enhanced environments. In the GenAI context, where feedback is abundant and learner-initiated, learners must actively interpret and evaluate AI suggestions (Dong, 2025). Engagement thus functions as the mechanism translating confidence into regulatory actions, highlighting its importance in instruction.

### Rethinking the role of self-efficacy in GenAI-assisted writing

The lack of a direct path from GenAI writing self-efficacy to most SRL strategies (metacognitive, social, motivational) after accounting for engagement warrants attention. In traditional contexts, self-efficacy often directly predicts strategy use (Teng, 2021; Tsao, 2021). These findings suggest that in GenAI-mediated environments, its influence may be more indirect and contingent on feedback engagement, indicating a shift in the relationship in technology-mediated writing. One explanation is that GenAI tools redistribute regulatory responsibility between learners and technology (Wang, 2024). High self-efficacy may encourage approaching GenAI with confidence (Liu and Zhang, 2025), but does not guarantee strategic use without deep feedback engagement. This highlights the need to reconceptualize motivational constructs in AI-supported learning, where learner-technology interaction mediates self-efficacy’s effects. Another explanation is that processing GenAI feedback requires new skills for effective engagement. For instance, prompt engineering skills are needed to obtain useful feedback. Without such skills, even learners with high self-efficacy may struggle to engage, limiting self-efficacy’s direct effects. This suggests GenAI writing self-efficacy involves confidence in both writing and interacting with GenAI tools.

### Theoretical implications

This study extends social cognitive theory of self-regulated learning (Bandura, 1997; Zimmerman and Kitsantas, 2014) by demonstrating feedback engagement’s mediating role between self-efficacy and SRL strategy use in GenAI-assisted writing. It shows engagement is both an outcome of self-efficacy and a key mediator translating beliefs into action, highlighting process-oriented variables. This study extends social cognitive theory and SRL theory by validating a process-oriented model in GenAI-assisted writing, identifying feedback engagement as a critical translational mechanism between self-efficacy and behavioral strategy use.

It also contributes to GenAI-assisted L2 writing research by focusing on psychological processes underlying learner-GenAI interaction. While prior studies often focus on writing outcomes or perceptions (Asadi et al., 2025; Chan et al., 2024; Liu and Zhang, 2025), this study examines relationships among key psychological constructs and their influence on writing processes. it advances the literature on L2 writing by revealing how motivational beliefs are converted into regulatory behaviors in AI-mediated environments, which was rarely examined in prior research.

Moreover, it enriches feedback engagement literature by exploring its multidimensional role in GenAI-assisted writing. Extending research beyond traditional teacher or peer feedback, it examines engagement with GenAI feedback, which is learner-initiated and iterative. The findings show its cognitive, behavioral, emotional, and ethical dimensions all contribute to mediation. It enriches feedback engagement theory by demonstrating its multidimensional and mediating role in GenAI contexts, going beyond traditional teacher feedback research.

### Practical implications

Instruction should explicitly target feedback engagement practices beyond technical tool training. Guidance can include critically evaluating AI feedback, comparing multiple responses, and making informed adoption decisions (Rad et al., 2024). Moreover, fostering GenAI writing self-efficacy should be paired with structured engagement opportunities. Activities like guided prompt revision and reflective logs can scaffold deeper engagement (Liu and Zhang, 2025). Teachers should also frame GenAI as a regulatory partner, not an authoritative source. Emphasizing learner agency can prevent over-reliance and promote sustainable self-regulated development. Curriculum designers could additionally incorporate GenAI-related skills into EFL writing courses, including prompt engineering, feedback evaluation, and strategy selection.

### Limitations and future research

This study relied exclusively on cross-sectional self-reported data, which reflect learners’ perceived self-efficacy, feedback engagement, and strategy use rather than directly observed writing behaviors. Thus, the findings may not fully represent actual engagement or strategic implementation during writing processes. Future studies may adopt mixed-methods approaches (e.g., screen recordings, think-aloud protocols, writing process tracking) to triangulate self-reports with objective behavioral evidence. What’s more, focusing on Chinese graduate EFL learners may limit generalizability. Further scholarly inquiry could validate the generalizability of the proposed model by testing it on learners of different demographics and varying proficiency levels. Moreover, modified existing scales were used, in which sense future research could develop new scales specifically for GenAI-related constructs to better capture unique characteristics.

## Conclusion

This study investigated the relationships among GenAI writing self-efficacy, engagement with GenAI feedback, and writing self-regulated learning strategies among graduate EFL writers. By testing a mediation model, the study demonstrated that engagement with GenAI feedback serves as the key mechanism through which self-efficacy is enacted in GenAI-assisted writing contexts. Specifically, GenAI writing self-efficacy significantly predicts feedback engagement, which in turn positively predicts all four dimensions of writing SRL strategies. The mediation effect is full for metacognitive, social, and motivational strategies, and partial for cognitive strategies.

The findings contribute to emerging theorization of AI-mediated L2 writing by highlighting the centrality of engagement as a process variable. Rather than assuming that confidence in using GenAI automatically leads to strategic writing behavior, this study shows that such beliefs must be realized through active, effortful engagement with feedback. In this sense, GenAI-assisted writing is best understood not as a shortcut to improved writing but as a context that foregrounds learners’ regulatory agency.

The practical implications of this study suggest that EFL writing instruction in GenAI-assisted contexts should focus on fostering feedback engagement practices, building GenAI writing self-efficacy, and framing GenAI as a regulatory partner. By doing so, teachers can help students develop the necessary skills and strategies to effectively use GenAI tools to support their self-regulated writing development.

Despite its limitations, this study provides important insights into the psychological processes underlying GenAI-assisted L2 writing and paves the way for future research in this rapidly evolving field. Future studies should address the limitations of this study and explore new directions to further advance our understanding of GenAI-assisted writing and its implications for L2 learning and teaching.

## Data Availability

The original contributions presented in the study are included in the article/supplementary material, further inquiries can be directed to the corresponding author.
